# Sports cardiology: lessons from the past and perspectives for the future

**DOI:** 10.12688/f1000research.6318.1

**Published:** 2015-04-20

**Authors:** Roman Leischik

**Affiliations:** 1 Faculty of Health, School of Medicine, Witten/Herdecke University, Hagen, 58095, Germany

**Keywords:** Cardiac Health, Negative remodelling, Sports screening, EECG, Myocardial Hypertrophy

## Abstract

The possibility of myocardial damage as a result of endurance sport has been known about since ancient times. According to a leg­end, a soldier named Pheidippides (more likely Philippides) dropped dead after run­ning from war-torn Marathon to Athens with the news of victory. Millennia later, we do not know whether he was a soldier or a courier, or whether he really ran the entire 240 km from Athens to Sparta and then back from Marathon to Athens. What is clear however, is that his death went down in history as the first documented exercise-related death and provides a tangible starting-point for the discipline of sport cardiology.

Sports cardiology today covers a broad range of areas; from patients with cardiomyopathies, coronary disease and metabolic syndrome through to fitness fans, high-performance athletes and those with physically demanding professions.

The following editorial introduces the primary topics for discussion to be included in the *F1000Research* channel Sports cardiology with the hope that this will evoke open, controversial and broad discourse in the form of reviews and original research papers in this important field.

## Editorial

Sport is of great social and medical significance. Accordingly, prevention of sudden cardiac sport-related deaths (SCD), prevention of negative cardiac remodelling or arrhythmias and training recommendations or rules for patients are extremely important to ensure wide-spread participation and the health benefits that this provides ^1^. The present collection of papers focusing on sport cardiology should stimulate lively, controversial, fair and future-oriented discussions.

### Structural changes in the athlete’s heart

Changes in the cardiac structures may occur as a consequence of repeated vigorous exercise. This adaptation of the heart to allow the accommodation of greater activity loads is a well-known phenomenon ^2^ and was first mentioned by Henschen, a Finnish physician, at the end of the 19 ^th^ century ^3^. One of the physiological modifications of the heart in response to sustained exercise is a ‘harmonious increase in size’ (also known as “healthy” myocardial hypertrophy ^4^. The influencing factors on the degree of hypertrophy include the kind of physical activity, individual genetic predisposition and environmental effects. Morganroth *et al.* ^5^ described in simplified terms that athletes who took part in endurance-based exercise would often present with eccentric hypertrophy as a result of prolonged and repeated volume overload. Conversely, the Morganroth hypothesis purported that athletes who underwent strength training were more likely to present with concentric hypertrophy. Today, it is recognised that there are more than two different types of athletic heart, the Morganroth hypothesis is not immediately applicable to all types of sports ^2^ and that more research is required into the extent and type of myocardial hypertrophy that can result from exercise.

### Sudden cardiac sports-related deaths (SCD), negative cardiac remodelling and the question of sport ‘dosage’.

The prevalence of sudden death in connection with sporting activity is about 4.6 people out of 10,000,000 per year in an average population. About 6% of this cohort comprises young athletes ^6^. Young competitive athletes have a 5-fold higher risk of sudden death than non-competitive athletes and men have a 20-fold higher risk than women *.* It is arguably more important however to pay attention to the variety of causes rather than to the absolute figures, which vary widely over the years and among studies ^7^.

Exercise-induced “cardiac fatigue” is a broadly discussed issue ^8– 10^, but one that still holds unanswered questions. Numerous investigations regarding the increase in biomarkers of left ventricular injury in endurance exercise/marathon ^11^ and triathlon ^11^ competitors have been conducted. Negative cardiac remodelling due by sporting activity can lead to arrhythmias ^12^ and atrial fibrilation ^13^. In this area the contribution of genetics must also be considered ^14^. The role of exercise-induced right ventricular injury is controversial and remains under discussion ^15, 16^.

Generally, endurance athletes and joggers ^17^ live longer compared to the general population ^10, 18^. The question of the intensity of physical activity and use of different methods of training in patients ^19^ and athletes ^20^ are potential themes of future studies. In the last 20 years many high-intensity interval training (HIT)-studies have been initiated ^20^, but as with any new exercise regime, the risks, advantages and exact definitions of a healthy ‘dose’ for different groups of patients ^19^ and athletes must be carefully defined through prospective investigation.

### Cardiac screening in sports

 The discussion about the extent and methods of screening in young/middle aged and old athletes/patients ^7, 21^ has been ongoing for years. The debate about screening examinations should consider not only SCD, but the possible cardiac structural changes caused by sport activities ^2, 8, 22, 23^ and implications for complications in long-term follow-up of athletes ^7, 23, 24^. Even in countries without sufficient public health systems, the costs for screening-examinations should be regarded as negligible given the high expenditures for preparation and participation in marathon and triathlon competitions ^25^ or intensive costs in professional football and other team sports. Inequalities in sports cardiology screening should not be a cause for natural selection or contribute to the possibility of later complications of aortic/atrial enlargement and arrhythmias. These complications can be seen in treatment centers as a major problem in long-term care with long-term follow-up. A discussion about global prices for sport screening should be initiated because of the importance of this examination for public health.

## Summary

There are a number of recurrent and salient topics in the field of sports cardiology: SCD in connection with sporting activity ^6^; cardiac “fatigue” ^8^ and cardiac injury caused by endurance sports ^10^; structural changes in an athlete’s heart ^2^ and negative cardiac remodelling ^9, 23^; screening methods for SCD ^21^ or cardiac remodelling ^7^; the right ‘dose’ of sport ^26^ and types of training methods ^20^. These have been briefly introduced in this Editorial, in the hope of stimulating research and discourse in these important areas, for which the channel ‘Sports cardiology’ will be a lively forum.
